# J-shaped association between the non-high-density lipoprotein cholesterol to high-density lipoprotein cholesterol ratio and uric acid in Chinese adults: A cross-sectional study from CHARLS

**DOI:** 10.1097/MD.0000000000050567

**Published:** 2026-09-18

**Authors:** Xiaodong Zhang, Jiaan Sun, Li Liu, Xiaopeng Li, Ruijie Xie, Yongfeng Yin, Lanjuan Xu

**Affiliations:** a Department of Neurology, Zhengzhou Central Hospital Affiliated to Zhengzhou University, Zhengzhou, China; b Department of Neurology, First Affiliated Hospital of Henan University, Kaifeng, China; c Department of Hand & Microsurgery, The Affiliated Nanhua Hospital, Hengyang Medical School, University of South China, Hengyang, China.

**Keywords:** CHARLS, cross-sectional study, HDL-C, HUA, NHHR, SUA, TC

## Abstract

The non-high-density lipoprotein cholesterol to high-density lipoprotein cholesterol ratio (NHHR) has emerged as a composite lipid indicator, but its association with serum uric acid (SUA) and hyperuricemia (HUA) remains insufficiently characterized, particularly in middle-aged and older Chinese adults. This cross-sectional study used baseline data from the China Health and Retirement Longitudinal Study (CHARLS). NHHR was calculated as (TC − HDL-C)/HDL-C. HUA was defined as SUA ≥ 7 mg/dL in men and ≥ 6 mg/dL in women. Survey-weighted multivariable linear and logistic regression models were used to examine associations of NHHR with SUA and HUA. Nonlinear relationships were explored using smooth curve fitting and two-piece linear regression models. Subgroup analyses and interaction tests were also conducted to investigate whether this relationship remains stable across different populations. A total of 11,132 participants aged ≥ 45 years were included. Higher NHHR was associated with higher SUA levels and greater odds of HUA in fully adjusted models. A nonlinear J-shaped association was observed between ln (NHHR) and SUA, with an inflection point at 1.49. Below this threshold, ln (NHHR) was inversely associated with SUA, whereas above it, the association became positive. A nonlinear pattern was also observed for HUA, although the association above the estimated threshold was not statistically significant. Subgroup analyses suggested potential effect modification by smoking and alcohol consumption; however, these findings were exploratory. In this cross-sectional analysis of middle-aged and older Chinese adults, NHHR was associated with SUA and HUA, and a J-shaped relationship was observed for SUA. These findings highlight a potential link between lipid imbalance and uric acid metabolism, which warrants further investigation in longitudinal and mechanistic studies.

## 1. Introduction

Hyperuricemia (HUA), typically defined as a serum uric acid (SUA) concentration of ≥ 7.0 mg/dL in men and ≥ 6.0 mg/dL in women, is a common metabolic abnormality and the biochemical basis of gout.^[1,2]^ In recent years, the prevalence of HUA has increased worldwide, making it an important public health concern.^[1,3,4]^ In addition to gout, elevated SUA levels have been associated with a range of cardiometabolic and renal disorders, including hypertension, chronic kidney disease, metabolic syndrome, type 2 diabetes, and nonalcoholic fatty liver disease.^[4–7]^ Notably, epidemiological studies have reported that HUA is associated with multiple cardiometabolic conditions; however, the temporal and causal relationships remain incompletely understood.^[8–10]^ Although these associations have been consistently reported, the underlying mechanisms linking uric acid metabolism with broader metabolic dysfunction remain incompletely understood.

NHHR is an emerging comprehensive lipid marker that integrates atherogenic (non-HDL-C) and antiatherogenic (HDL-C) lipoprotein components, thereby providing a more comprehensive assessment of cardiovascular and metabolic disease risk.^[11–13]^ Non-HDL-C includes all pro-atherosclerotic cholesterol, such as low-density lipoprotein cholesterol (LDL-C), very low-density lipoprotein (VLDL), and their remnants, while HDL-C has protective effects such as anti-inflammatory, antioxidant, and cholesterol reverse transport properties.^[11,14,15]^ Recent studies have further confirmed the superiority of NHHR in assessing the risk of various diseases, including coronary artery disease, diabetes, chronic kidney disease, and cerebrovascular disease.^[16–19]^ The predictive ability of NHHR may stem from a more accurate reflection of lipid metabolism imbalance, whereas testing non-HDL-C or HDL-C alone may not fully capture this imbalance.

Although studies in the United States have examined the relationship between NHHR and HUA, large-scale data in Chinese populations are lacking. Using data from the 2011 CHARLS, this study investigates the association between NHHR and SUA and HUA in Chinese adults. Although previous studies have explored the associations of NHHR or related lipid markers with hyperuricemia in other populations, evidence from nationally representative samples of middle-aged and older Chinese adults remains limited. The present study extends existing literature by evaluating both linear and nonlinear associations in CHARLS.

## 2. Methods

### 2.1. Study population

The data for this study were obtained from the CHARLS. The national baseline survey (first wave) conducted in 2011 covered 150 counties and districts in 28 provinces and 450 villages in China, with a total of 17,708 middle-aged and elderly residents aged 45 and above included in the study. Data were collected via computer-assisted personal interviews (CAPI), covering diverse information such as demographic characteristics, family structure, health status, medical insurance, economic status, and biomarkers. The study protocol was approved by the Beijing University Biomedical Ethics Committee (IRB00001052-11015), and all participants provided informed consent. Raw data are publicly available through the CHARLS official website (http://charls.pku.edu.cn).^[20]^ We excluded participants with NHHR values outside the range of 1 to 20 to minimize the influence of implausible extreme values and potentially unstable ratios, which may arise from measurement error or very low HDL-C concentrations. This restriction was intended to improve model stability.

**Figure 1.**
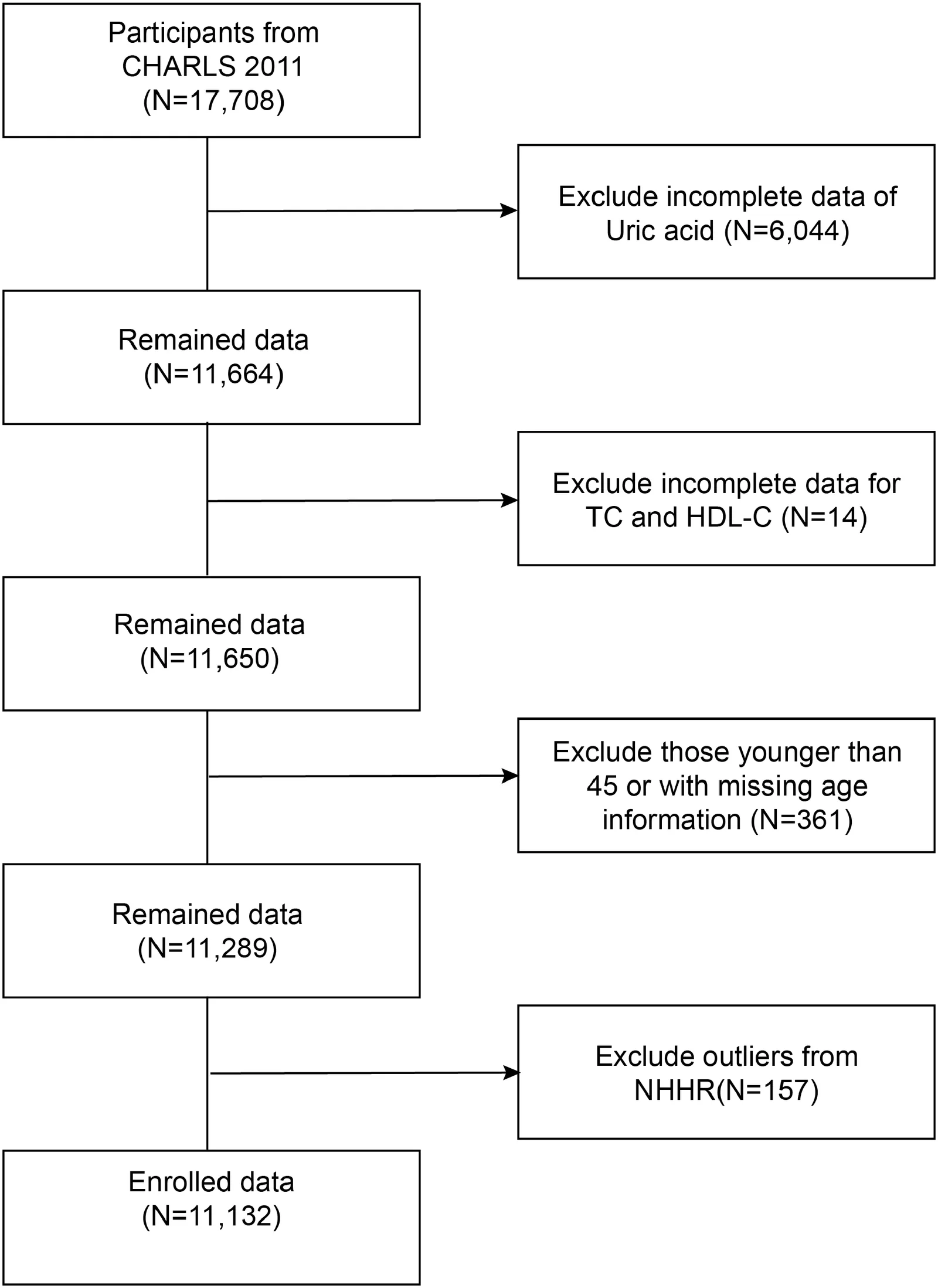
Flow chart of participant selection. CHARLS, China Health and Retirement Longitudinal Study.

This study utilized data collected in 2011 (Wave 1). The sample size for Wave 1 was 17,708 individuals. We excluded 6576 cases due to the following reasons: lack of uric acid information; lack of total cholesterol information and high-density lipoprotein cholesterol; age < 45 years or missing age information; NHHR values outside the range of 1 ≤ NHHR ≤ 20. The cross-sectional analysis included 11,132 participants. The detailed flowchart of the selection process is shown in Figure 1.

### 2.2. Definition of exposure and outcome

This study used NHHR as the exposure variable. Total cholesterol (TC) and high-density lipoprotein cholesterol (HDL-C) were quantified using an enzymatic colorimetric method. Fasting venous blood samples were collected during standardized health examinations, processed within 1 to 2 hours, and stored at − 20°C before centralized laboratory analyses conducted by the Chinese Center for Disease Control and Prevention (CDC) using standardized protocols with strict quality control procedures. SUA was measured using the uricase-peroxidase method. Non-HDL-C was calculated as TC minus HDL-C, and NHHR was defined as (TC − HDL-C)/ HDL-C. The primary outcomes were SUA and HUA, defined as SUA ≥ 7 mg/dL in males and ≥ 6 mg/dL in females.^[4,12]^

### 2.3. Covariables

Based on previous research,^[21]^ we adjusted for the following potential confounders in the multivariable analyses: demographic characteristics (age, modeled as a continuous variable in years, and sex [male/female]); medical history (hypertension, diabetes, kidney disease, liver disease, and stroke, each coded dichotomously as yes/no); lifestyle factors (smoking status and alcohol consumption, both coded as yes/no); anthropometric measurement (body mass index [BMI, kg/m^2^], categorized according to World Health Organization criteria as < 25.0 [normal], 25.0–29.9 [overweight], and ≥ 30.0 [obese])^[22]^; and biochemical indicators (triglyceride–glucose [TyG] index, glycated hemoglobin, serum creatinine, and cystatin C). All covariates were simultaneously included in the fully adjusted multivariable models.

### 2.4. Statistical analysis

Baseline characteristics of participants were compared across NHHR quartiles using survey-weighted linear regression for continuous variables and weighted chi-square tests for categorical variables. Survey-weighted multivariable linear and logistic regression models were used to examine the associations of NHHR with SUA and HUA, respectively. Three models were constructed: Model 1 was unadjusted; Model 2 was adjusted for age and sex; and Model 3 was fully adjusted for age, sex, hypertension, diabetes, kidney disease, liver disease, stroke, smoking status, alcohol consumption, BMI, triglyceride–glucose (TyG) index, glycated hemoglobin, creatinine, and cystatin C.

NHHR was analyzed both as a continuous variable and as quartiles, and tests for trend were conducted by assigning the median value of each quartile as a continuous variable in the models. Subgroup analyses were performed to evaluate potential effect modification by age, sex, BMI category, hypertension, diabetes, stroke, kidney disease, and liver disease, and interaction tests were conducted by including multiplicative interaction terms in the models. To explore potential nonlinear relationships, smooth curve fitting based on generalized additive models was applied.

Missing data were handled as follows: participants with missing data on the exposure (NHHR) or outcomes (SUA and HUA) were excluded from the analysis, while missing covariate values were imputed using simple imputation methods. Specifically, mean imputation was applied for variables with approximately normal distributions, whereas median (50th percentile) imputation was used for skewed variables. All analyses were conducted using R (version 4.2) and EmpowerStats (version 5.0), with statistical significance defined as a two-sided *P* < .05.

## 3. Results

### 3.1. Baseline characteristics

Among the 11,132 participants aged 45 years or older, the mean (SD) age was 59.46 (9.49) years, and 53.13% were female. The mean (SD) serum uric acid (SUA) concentration was 4.47 (1.26) mg/dL, and 644 participants (5.79%) met the definition of hyperuricemia. Stratification by NHHR quartiles showed that participants in higher quartiles had less favorable metabolic profiles: higher BMI, SUA, total cholesterol, triglycerides, triglyceride–glucose index (TYG), glycated hemoglobin, creatinine, and cystatin C, and lower HDL-C (all *P* for trend < .001). The prevalence of hypertension increased across quartiles (Q1: 18.90%; Q4: 36.54%; *P* < .001), as did diabetes (Q1: 3.38%; Q4: 10.49%; *P* < .001), hyperuricemia (Q1: 3.34%; Q4: 10.42%; *P* < .001), and stroke (Q1: 1.80%; Q4: 3.99%; *P* < .001). In contrast, kidney disease and liver disease did not differ significantly across quartiles (*P* = .886 and *P* = .322, respectively) (Table 1).

**Table 1:** Baseline characteristics of participants according to the quartiles of NHHR.

Characteristics	NHHR				*P*-value
	Q1 (N = 2783)	Q2 (N = 2783)	Q3 (N = 2783)	Q4 (N = 2783)	
Age (years)	59.89 ± 9.81	59.52 ± 9.84	59.10 ± 9.13	59.32 ± 9.17	.063
Gender, (%)					<.001
Male	50.23	45.63	45.06	46.57	
Female	49.77	54.37	54.94	53.43	
Hypertension, (%)					<.001
Yes	18.90	24.65	28.93	36.54	
No	81.10	75.35	71.07	63.46	
Diabetes, (%)					<.001
Yes	3.38	4.78	6.43	10.49	
No	96.62	95.22	93.57	89.51	
Stroke, (%)					<.001
Yes	1.80	2.41	2.77	3.99	
No	98.20	97.59	97.23	96.01	
Kidney Diseases, (%)					.886
Yes	6.14	5.71	5.79	5.71	
No	93.86	94.29	94.21	94.29	
Liver Diseases, (%)					.322
Yes	3.99	3.34	3.27	3.16	
No	96.01	96.66	96.73	96.84	
Current smoking, (%)					<.001
Yes	31.69	28.46	27.96	26.19	
No	68.31	71.54	72.04	73.81	
Current alcohol use, (%)					<.001
Yes	38.70	32.59	29.75	28.78	
No	61.30	67.41	70.25	71.22	
BMI (Kg/m^2^)	22.36 ± 13.68	23.90 ± 46.14	24.26 ± 12.03	25.08 ± 11.01	<.001
SUA (mg/dl)	4.25 ± 1.20	4.31 ± 1.20	4.48 ± 1.22	4.85 ± 1.34	<.001
Glycated Hemoglobin, (%)	5.12 ± 0.60	5.20 ± 0.67	5.29 ± 0.80	5.47 ± 1.08	<.001
Cystatin C	1.04 ± 0.29	1.03 ± 0.25	1.00 ± 0.21	0.99 ± 0.26	<.001
Creatinine (mg/dl)	0.77 ± 0.33	0.78 ± 0.19	0.78 ± 0.18	0.81 ± 0.21	<.001
TYG	8.22 ± 0.45	8.50 ± 0.47	8.78 ± 0.49	9.30 ± 0.66	<.001
HUA, (%)					<.001
Yes	3.34	4.20	5.17	10.42	
No	96.66	95.80	94.83	89.58	

Mean ± SD for continuous variables: the *P* value was calculated by the weighted linear regression model; for categorical variables: the *P* value was calculated by the weighted chi-square test.

BMI = body mass index, HUA = hyperuricemia, Q = quartile, SUA = serum uric acid, TYG = triglyceride Glycaemic Index.

### 3.2. Association between NHHR and SUA and HUA

Table 2 shows the associations of ln(NHHR) with SUA and HUA. In the fully adjusted model (Model 3), each 1-unit increase in ln(NHHR) was associated with 0.29 mg/dL higher SUA [β = 0.29; 95% CI: 0.23–0.35; *P* < .001] and 1.75-fold higher odds of HUA prevalence [OR = 1.75; 95% CI: 1.33–2.30; *P* < .001]. When ln(NHHR) was categorized into quartiles, participants in Q4 had 0.33 mg/dL higher SUA [β = 0.33; 95% CI: 0.26–0.40; *P* < .001] and 1.75 times higher odds of HUA prevalence [OR = 1.75; 95% CI: 1.29–2.38; *P* < .001] compared with Q1. All models showed a consistent dose–response trend (P for trend < 0.001).

**Table 2:** Relationship between NHHR and SUA and HUA.

Ln(NHHR)	SUA	HUA
	β (95% CI)	OR (95% CI)
Crude model (Model 1)		
Continuous	0.52 (0.47, 0.58)	3.23 (2.67, 3.91)
Categories		
Quartile 1	0 (ref)	1 (ref)
Quartile 2	0.05 (−0.01, 0.12)	1.27 (0.96, 1.68)
Quartile 3	0.23 (0.16, 0.29)	1.58 (1.21, 2.06)
Quartile 4	0.60 (0.53, 0.66)	3.36 (2.65, 4.28)
P for trend	<0.001	<0.001
Minimally adjusted model (Model 2)		
Continuous	0.57 (0.52, 0.62)	3.40 (2.81, 4.12)
Categories		
Quartile 1	0 (ref)	1 (ref)
Quartile 2	0.10 (0.04, 0.16)	1.31 (0.99, 1.73)
Quartile 3	0.29 (0.23, 0.35)	1.68 (1.28, 2.19)
Quartile 4	0.64 (0.58, 0.70)	3.56 (2.80, 4.54)
P for trend	<0.001	<0.001
Fully adjusted model (Model 3)		
Continuous	0.29 (0.23, 0.35)	1.75 (1.33, 2.30)
Categories		
Quartile 1	0 (ref)	1 (ref)
Quartile 2	0.02 (−0.03, 0.07)	1.05 (0.78, 1.42)
Quartile 3	0.14 (0.08, 0.20)	1.24 (0.93, 1.67)
Quartile 4	0.33 (0.26, 0.40)	1.75 (1.29, 2.38)
P for trend	<0.001	<0.001

Model 1: No covariates adjusted. Model 2: Adjusted for age and gender. Model 3: Adjusted for age, sex, hypertension, diabetes, kidney disease, liver disease, stroke, smoking, alcohol consumption, BMI, TYG, glycated hemoglobin, creatinine, cystatin C.

BMI = body mass index, HUA = hyperuricemia, Q = quartile, SUA = serum uric acid, TYG = Triglyceride Glycaemic Index.

**Figure 2.**
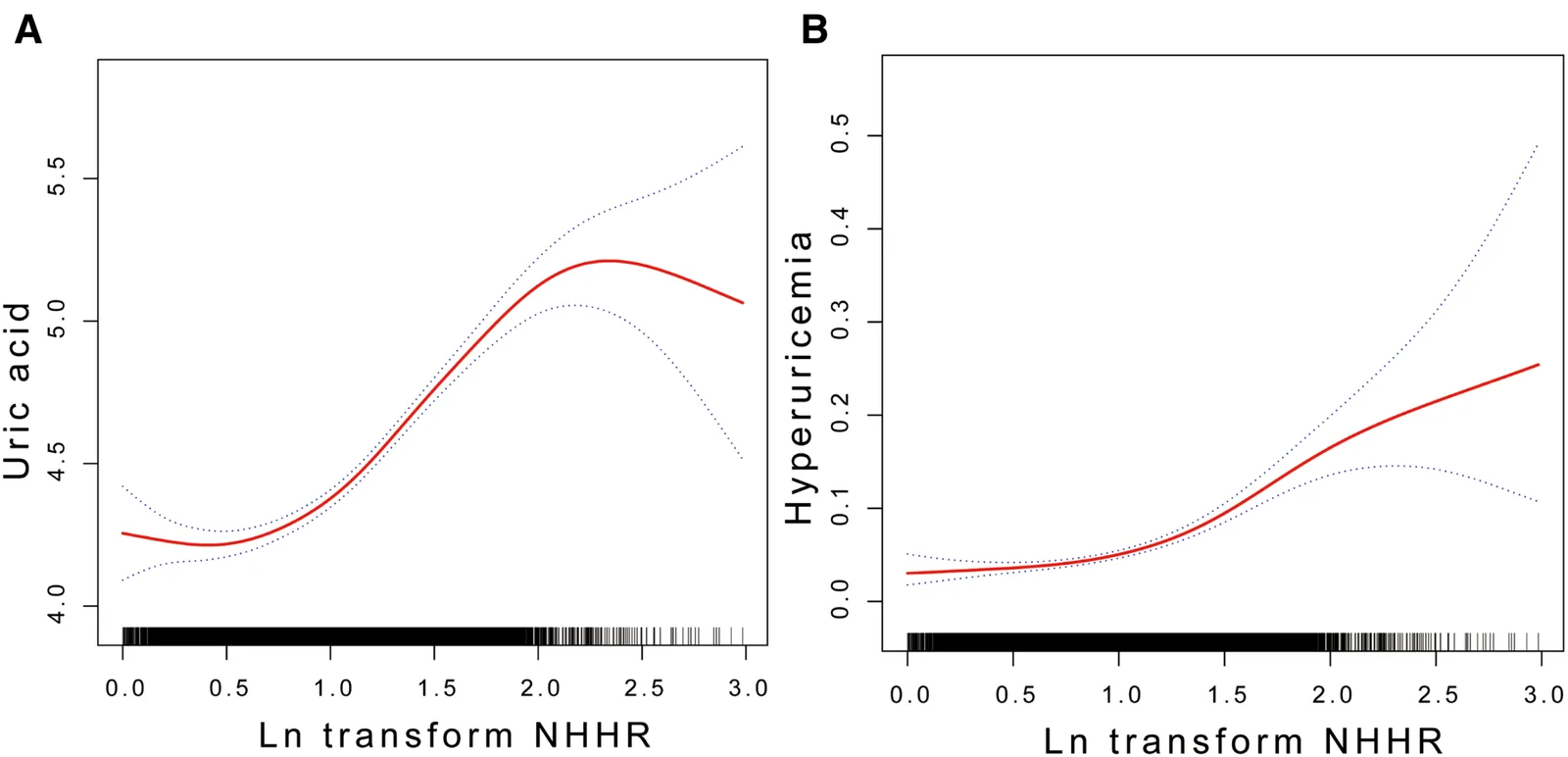
Nonlinear association between NHHR and uric acid. The solid red curve represents the smooth curve fit between the variables. The black band indicates the 95% confidence interval of the fit results. (A) Ln(NHHR) and SUA, (B) Ln(NHHR) and HUA.

Furthermore, smooth curve fitting and threshold effect analyses revealed a J-shaped nonlinear relationship between ln(NHHR) and SUA, with a significant inflection point at ln(NHHR) = 1.49 (Table 3, Fig. 2). Below this point, ln(NHHR) was inversely associated with SUA [β = −0.58; 95% CI: −0.91 to − 0.24; *P* < .01], whereas above this point, ln(NHHR) was positively associated with SUA [β = 0.11; 95% CI: 0.09–0.13; *P* < .01]. A nonlinear association was also observed for HUA; however, the estimated threshold occurred at a relatively high ln(NHHR) value and should therefore be interpreted with caution, particularly given the limited clinical plausibility and the nonsignificant association above the threshold.

**Table 3:** The relationship between NHHR and SUA and HUA was analyzed using a two-stage regression model.

Adjusted model	SUA [β (95%CI)]	HUA [OR (95%CI)]
**Model 1**	0.10 (0.08, 0.12) < 0.01	1.16 (1.08, 1.24) < 0.01
**Model 2**		
Inflection point (K)	1.49	5.67
< K	−0.58 (−0.91, −0.24) < 0.01	1.22 (1.12, 1.34) < 0.01
≥ K	0.11 (0.09, 0.13) < 0.01	1.01 (0.86, 1.19) 0.87
Log-likelihood ratio test	<0.01	0.08

BMI = body mass index, CI = confidence interval, HUA = hyperuricemia, Q = quartile, SUA = serum uric acid, TYG = Triglyceride Glycaemic Index.

Adjusted for age, sex, hypertension, diabetes, kidney disease, liver disease, stroke, smoking, alcohol consumption, BMI, TYG, glycated hemoglobin, creatinine, cystatin C.

### 3.3. Subgroup analyses

Subgroup analyses were conducted to evaluate the robustness of the associations between NHHR and SUA as well as HUA. As shown in Table 4, alcohol consumption and smoking status significantly modified the association between NHHR and SUA (both *P* for interaction < .001). Specifically, NHHR exhibited a markedly stronger positive association with SUA among nondrinkers [β = 0.38; 95% CI: 0.30–0.45; *P* < .01] compared with current drinkers [β = 0.13; 95% CI: 0.03–0.23; *P* < .01]. Similarly, the association was stronger among nonsmokers [β = 0.36; 95% CI: 0.29–0.44; *P* < .01] than among current smokers [β = 0.12; 95% CI: 0.02–0.23; *P* < .01]. The observed differences across smoking and alcohol subgroups should be interpreted cautiously because subgroup analyses were exploratory and may be susceptible to chance findings. No statistically significant interactions were observed for sex, age, BMI, hypertension, diabetes, stroke, kidney disease, or liver disease (all *P* for interaction > .05), indicating that the positive association between NHHR and SUA was generally consistent across these subgroups.

**Table 4:** Subgroup analysis of the relationship between NHHR and Uric acid.

Subgroup	SUA [β (95%CI)]	*P* for interaction
Sex		.057
Male	0.23 (0.15, 0.32)	
Female	0.35 (0.26, 0.44)	
Age		.119
< 60 years	0.33 (0.25, 0.42)	
≥ 60 years	0.24 (0.14, 0.33)	
BMI (Kg/m^2^)		.890
< 24.9 kg/m^2^	0.25 (0.17, 0.32)	
25–29.9 kg/m^2^	0.28 (0.15, 0.42)	
≥ 30 kg/m^2^	0.25 (−0.08, 0.57)	
Hypertension, (%)		.973
Yes	0.29 (0.17, 0.41)	
No	0.29 (0.22, 0.36)	
Diabetes, (%)		.998
Yes	0.28 (0.03, 0.53)	
No	0.28 (0.22, 0.35)	
Stroke, (%)		.365
Yes	0.48 (0.07, 0.88)	
No	0.28 (0.22, 0.35)	
Kidney Diseases, (%)		.663
Yes	0.21 (−0.44, 0.46)	
No	0.27 (0.21, 0.33)	
Liver Diseases, (%)		.065
Yes	−0.03 (−0.38, 0.32)	
No	0.30 (0.24, 0.37)	
Current alcohol use, (%)		**<.001**
Yes	0.13 (0.03, 0.23)	
No	0.38 (0.30, 0.45)	
Current smoking, (%)		**<.001**
Yes	0.12 (0.02, 0.23)	
No	0.36 (0.29, 0.44)	

Adjusted for age, sex, hypertension, diabetes, kidney disease, liver disease, stroke, smoking, alcohol consumption, BMI, TYG, glycated hemoglobin, creatinine, cystatin C.

BMI = body mass index, HUA = hyperuricemia, Q = quartile, SUA = serum uric acid, TYG = Triglyceride Glycaemic Index.

Consistent patterns were observed for HUA (Table 5). Alcohol consumption significantly modified the association between NHHR and HUA (*P* for interaction < .001), with a stronger association observed among current drinkers [OR = 2.06; 95% CI: 1.45–2.95] compared with nondrinkers [OR = 1.45; 95% CI: 0.95–2.21]. However, no significant interactions were detected for sex, age, BMI, hypertension, diabetes, stroke, kidney disease, liver disease, or smoking status (all *P* for interaction > .05). Overall, these findings indicate that the association between NHHR and HUA remained largely stable across most demographic and clinical subgroups, except for alcohol consumption.

**Table 5:** Subgroup analysis of the relationship between NHHR and HUA.

Subgroup	HUA [OR (95% CI)]	*P* for interaction
Sex		.291
Male	1.55 (1.10, 2.20)	
Female	2.05 (1.34, 3.15)	
Age		.885
< 60 years	1.82 (1.22, 2.70)	
≥ 60 years	1.71 (1.18, 2.48)	
BMI (Kg/m^2^)		.583
< 24.9 kg/m^2^	1.67 (1.21, 2.32)	
25–29.9 kg/m^2^	1.46 (0.83, 2.56)	
≥ 30 kg/m^2^	2.82 (0.93, 8.56)	
Hypertension, (%)		.815
Yes	1.65 (1.07, 2.54)	
No	1.78 (1.26, 2.51)	
Diabetes, (%)		.260
Yes	3.06 (1.07, 8.69)	
No	1.67 (1.26, 2.22)	
Stroke, (%)		.897
Yes	1.88 (0.41, 8.49)	
No	1.75 (1.33, 2.31)	
Kidney Diseases, (%)		.156
Yes	0.86 (0.31, 2.39)	
No	1.86 (1.40, 2.47)	
Liver Diseases, (%)		.793
Yes	2.31 (0.30, 17.83)	
No	1.78 (1.35, 2.34)	
Current alcohol use, (%)		**<.001**
Yes	2.06 (1.45, 2.95)	
No	1.45 (0.95, 2.21)	
Current smoking, (%)		.794
Yes	1.65 (1.02, 2.67)	
No	1.82 (1.31, 2.53)	

Adjusted for age, sex, hypertension, diabetes, kidney disease, liver disease, stroke, smoking, alcohol consumption, BMI, TYG, glycated hemoglobin, creatinine, cystatin C.

BMI = body mass index, HUA = hyperuricemia, Q = quartile, SUA = serum uric acid, TYG = Triglyceride Glycaemic Index.

## 4. Discussion

In this cross-sectional study including 11,132 middle-aged and older Chinese adults, we observed a significant positive association of NHHR with serum uric acid (SUA) levels and the prevalence of hyperuricemia (HUA) after adjustment for demographic characteristics, lifestyle factors, comorbidities, BMI, and relevant biochemical indicators. This association remained generally consistent when ln(NHHR) was modeled as a continuous variable and when it was categorized into quartiles. Further smooth curve fitting and two-piece linear regression analyses suggested a potential J-shaped nonlinear association between ln(NHHR) and SUA, with an estimated inflection point of 1.49. Subgroup analyses indicated that smoking status and alcohol consumption might modify the association between NHHR and SUA, whereas the association between NHHR and HUA was generally stable across most subgroups, except for a potential interaction with alcohol consumption. Overall, these findings suggest a possible link between lipid metabolic imbalance and abnormal uric acid metabolism. However, given the cross-sectional design, the results should be interpreted as epidemiological associations rather than evidence of causality.

Previous studies have reported associations of NHHR with HUA or related metabolic outcomes. For example, Wang et al^[21]^ observed a positive association between NHHR and HUA, whereas He et al^[23]^ reported a potential nonlinear association between NHHR and HUA in cancer patients using NHANES data. Consistent with these findings, our study also observed positive associations of NHHR with both SUA and HUA. It should be emphasized that the present study was not intended to propose a new pathophysiological concept, nor can it establish a novel causal pathway linking NHHR to uric acid metabolism. Rather, the main value of this study lies in providing external validation and population-specific evidence for previously reported associations and potential nonlinear patterns between NHHR and abnormal uric acid metabolism in a nationally representative sample of middle-aged and older Chinese adults from CHARLS. Therefore, our findings should be interpreted as a supplement to, and validation of, existing evidence rather than as a substantial change in the conceptual understanding of this relationship.

The J-shaped association observed in this study and the estimated inflection point of ln (NHHR) = 1.49 represent noteworthy findings, but they should be interpreted with caution. First, this inflection point was estimated from statistical models and should not be regarded as a validated clinical threshold or intervention cutoff. When converted to the original NHHR scale, ln (NHHR) = 1.49 corresponds approximately to an NHHR value of 4.44. However, this value only describes the nonlinear pattern observed in the present dataset and does not imply clear clinical decision-making significance. Second, the positive association between NHHR and SUA above the inflection point is biologically plausible, as higher NHHR generally reflects a greater atherogenic lipid burden and relatively lower HDL-C-related protective capacity, both of which may coexist with metabolic dysfunction, insulin resistance, and impaired uric acid metabolism.^[12,24]^ However, the inverse association observed below the inflection point is more difficult to explain and may be related to nutritional status, underlying disease patterns, unmeasured confounding, sparse observations in the lower NHHR range, or model instability at the distributional tail. Furthermore, although smooth curve fitting and two-piece linear regression were used to explore potential nonlinearity, no dedicated sensitivity analysis was performed to validate the stability of this inflection point. Therefore, the J-shaped relationship should be considered exploratory and requires further validation in other populations and longitudinal studies.

The subgroup findings also require cautious interpretation. We observed statistically significant interactions of smoking status and alcohol consumption with the association between NHHR and SUA, with a stronger positive association among nonsmokers and nondrinkers. This pattern may suggest that, in the absence of smoking- or alcohol-related metabolic influences, the relationship between lipid metabolic imbalance and abnormal uric acid metabolism may be more apparent. Conversely, smoking and alcohol consumption may affect lipid metabolism, oxidative stress, hepatic and renal metabolic function, and SUA levels through multiple pathways. They may also cluster with dietary patterns, physical activity, socioeconomic status, and other health-related behaviors.^[25–27]^ Therefore, the observed subgroup differences may reflect competing metabolic effects, residual confounding, selection bias, differences in lipid metabolism, or behavioral clustering, rather than true biological effect modification. Importantly, multiple subgroup and interaction analyses were performed in this study without correction for multiple comparisons; therefore, these interaction findings should be regarded as exploratory and should not be interpreted as definitive evidence of effect modification.

From a biological perspective, the association between NHHR and SUA/HUA may reflect the shared involvement of lipid metabolic disturbance, insulin resistance, abnormal renal urate handling, and low-grade inflammation. NHHR integrates information from non-HDL-C and HDL-C, thereby reflecting both atherogenic lipoprotein burden and relative insufficiency of antiatherogenic lipoproteins.^[4,11,28,29]^ Thus, NHHR may better capture overall lipid metabolic imbalance than a single lipid parameter. Previous studies have suggested that insulin resistance may be associated with reduced renal urate excretion and elevated SUA levels, while lower HDL-C and higher concentrations of atherogenic lipoproteins may be related to oxidative stress and low-grade inflammation.^[29]^ However, insulin resistance indices, inflammatory biomarkers, oxidative stress markers, and renal urate transporter-related proteins such as URAT1 and ABCG2 were not directly measured in the present study. Therefore, these explanations should be regarded only as biologically plausible hypotheses based on previous literature, rather than mechanisms directly demonstrated by our data. The present findings support only a statistical association between NHHR and abnormal uric acid metabolism and do not establish specific molecular or physiological pathways.

Reverse causality is another important issue that should be considered when interpreting our findings. Although higher NHHR may be associated with elevated SUA through metabolic dysfunction, elevated SUA itself may also contribute to the development of lipid metabolic abnormalities. Previous studies have suggested that higher uric acid levels may be associated with oxidative stress, endothelial dysfunction, chronic low-grade inflammation, and metabolic dysregulation, which may further influence non-HDL-C, HDL-C, and their ratio. Therefore, the association between NHHR and SUA/HUA may be bidirectional, or it may arise from shared upstream metabolic risk factors such as obesity, insulin resistance, impaired renal function, dietary patterns, or other metabolic disturbances. Because this study was based on cross-sectional data collected at a single time point, we cannot determine whether increased NHHR preceded elevated SUA or whether elevated SUA contributed to altered lipid metabolism. Longitudinal studies with repeated measurements of lipid components and SUA are needed to clarify the temporal sequence and potential directionality of this relationship.

In terms of practical implications, our findings suggest that NHHR may be associated with abnormal uric acid metabolism, but they do not currently support the direct use of NHHR as a screening, diagnostic, prognostic, or clinical decision-making tool for HUA. The present study did not evaluate the predictive performance of NHHR for HUA, nor did it conduct receiver operating characteristic curve analysis, calibration analysis, reclassification analysis, or decision curve analysis. Therefore, our findings cannot be used to infer that NHHR provides incremental predictive value beyond traditional risk factors, nor do they justify the use of a specific NHHR threshold for clinical screening or intervention. A more appropriate interpretation is that NHHR, as an easily obtainable composite lipid indicator, may help improve epidemiological understanding of the relationship between lipid metabolic imbalance and abnormal uric acid metabolism. Future studies investigating its clinical utility should further assess its incremental predictive value, stability across repeated measurements, and external validity in different populations.

This study has several strengths. First, it was based on CHARLS data, included a relatively large sample size, and used a multistage stratified sampling design, which may better reflect the characteristics of middle-aged and older adults in China. Second, this study evaluated the associations of NHHR with both continuous SUA levels and the categorical outcome of HUA, and further explored linear, categorical, and nonlinear associations. Third, the multivariable models included demographic characteristics, lifestyle factors, comorbidities, BMI, and several biochemical indicators, which reduced the potential influence of confounding to some extent.

However, several important limitations should also be acknowledged. First, because of the cross-sectional design, the temporal relationship between NHHR and SUA/HUA could not be determined, and reverse causality could not be excluded. Second, residual confounding is one of the most important limitations of this study. Although we adjusted for various demographic, lifestyle, clinical, and biochemical covariates, CHARLS did not provide, or did not sufficiently provide, information on several factors that are closely related to both NHHR and SUA levels, including urate-lowering therapy, lipid-lowering medications such as statins and fibrates, renal disease severity, dietary purine intake, fructose consumption, metabolic syndrome severity, menopausal status, and more detailed socioeconomic factors. Medication use and dietary exposures are particularly important because they may directly alter lipid profiles or uric acid concentrations. Therefore, the influence of residual confounding may be substantial rather than merely theoretical, and the observed associations should not be interpreted as independent causal effects. Third, some covariates were obtained from self-reported questionnaires, which may have introduced recall bias or misclassification. Fourth, simple imputation was used to handle missing covariate data, which may have introduced bias and underestimated the uncertainty of the estimates. Finally, the J-shaped inflection point was derived from the present dataset and model estimation and has not been validated using an independent dataset or dedicated sensitivity analyses; therefore, its stability and clinical relevance require further confirmation.

In summary, this study observed positive associations of NHHR with SUA levels and the prevalence of HUA among middle-aged and older Chinese adults, and further suggested a potential J-shaped nonlinear association between NHHR and SUA. However, these findings should be regarded as exploratory and hypothesis-generating. Given the cross-sectional design, potential residual confounding, possible reverse causality, and lack of predictive performance assessment, the results should not be overinterpreted as evidence of causality or direct clinical applicability. Future prospective cohort studies and mechanistic investigations are warranted to validate the temporal relationship, potential mechanisms, and clinical relevance of NHHR in abnormal uric acid metabolism.

## 5. Conclusion

In this cross-sectional study of middle-aged and older Chinese adults, NHHR was associated with SUA levels and HUA prevalence, and a J-shaped relationship was observed between NHHR and SUA. These findings should be considered exploratory and hypothesis-generating. Prospective studies are warranted to clarify the temporal relationship and potential clinical relevance of NHHR in uric acid metabolism.

## Acknowledgments

We would like to thank all participants in this study.

## Author contributions

**Formal analysis:** Xiaodong Zhang.

**Supervision:** Jiaan Sun, Lanjuan Xu.

**Data curation:** Li Liu.

**Writing – original draft:** Xiaodong Zhang, Xiaopeng Li.

**Writing – review & editing:** Ruijie Xie.

**Methodology:** Yongfeng Yin.
